# High Velocity Bullets

**Published:** 1899-12-09

**Authors:** 


### HIGH VELOCITY BULLETS.

Dr. Keith and Mr. Rigby,[1] of the London Hospital, have been making some interesting experiments as to the effects produced by the bullets now being used in South Africa, and also by those bare-nosed and hollow-nosed projectiles which go by the name of Dum-Dum and Mark IV. bullets. It is to be noted that, although the Mauser is somewhat smaller than the Lee-Metford bullet, its velocity is somewhat higher, so that the momentum is about the same in each case, and thus any difference in their effects depends principally upon difference in their expansibility. In performing these experiments care was taken to select a cadaver in which the amount of fluid in the tissues seemed to represent, as nearly as might be, the amount present during life, and also in which the tissues had been preserved by the use of a solution by which they were rendered sufficiently firm to enable a section to be made afterwards, so as to ascertain with some degree of exactitude the amount of destruction which had been produced.

In comparing the destructive effects of bullets on the human body "three sharply differentiated types of wounds have to be distinguished: (1) Pure flesh wounds; (2) wounds in which bone is involved, and crushing effects are produced; and (3) wounds in which the bullet has passed through a cavity filled with fluid contents—*e.g.*, the capsule of the liver containing blood and hepatic substance, the medullary canal of a long bone containing marrow, or the skull containing brain and blood." It is to be noted in regard to all these injuries that neither the wound of entrance nor of exit is any index to the amount of destruction wrought. The entrance and exit wounds of fully-mantled bullets are almost invariably equal to their diameter, but the destructive track within is much wider, yet although arteries, tendons, and nerves situated within the line of the bullet's course are cut through, those outside that axis and yet within the area of destruction seem to be left practically uninjured. The bullet meets with such little resistance in passing through the soft parts that it can unburden itself of but little energy, and thus can work but little destruction unless it be expansile or moving with a wide spin. But the results are quite different when a bullet meets with a bone. It crashes into it and carries before it the fragments, to which it imparts much of its own velocity, driving them through the softer and less resistant tissues. It matters little what sort of a bullet is used if bone be once struck. All small-bore bullets can under such circumstances work damage absolutely fatal to the future of a limb.

In regard to the third class of injuries produced by bullets of high velocity—viz., those marked by "explosive effects"—it is to be noted that, although for their production the cavity need not be closed, it must have its contents in a state of fluidity. There is no doubt that these so-called explosive effects occur much more markedly with a flat-nosed bullet like the Dum-Dum than with a fully-mantled bullet, but any bullet will produce them if its velocity is great enough. In fact, every bullet wound is attended with more or less explosive effect. Even in firing at sections of the thigh it was found that not only the marrow in the bones but the clots in the arteries and veins were driven out at the cut ends, and, in reference to the shattering of the cranium produced by bullets, it is stated that within the first hundred yards of their course the momentum of any of the modern military bullets is sufficient to burst the skull open even were it five times its normal strength. The writers of this paper, however, agree with Professor Ogston that Von Bruns has overstated the case against the English open-nosed bullets. In the first place, the Dum-Dum bullets which he used were much exaggerated forms; secondly, the wounds which he figured in his papers dealing with the effect of the Dum-Dum and the

Mark IV. bullets were such as these observers have been unable to reproduce; and thirdly, the wounds were greatly exaggerated from the fact that the dried and wizened legs of cadavers were mostly used in the experiments.

[1] Lancet, Dec. 2.

